# Assembly of the MHC I peptide-loading complex determined by a conserved ionic lock-switch

**DOI:** 10.1038/srep17341

**Published:** 2015-11-27

**Authors:** Andreas Blees, Katrin Reichel, Simon Trowitzsch, Olivier Fisette, Christoph Bock, Rupert Abele, Gerhard Hummer, Lars V. Schäfer, Robert Tampé

**Affiliations:** 1 Institute of Biochemistry, Biocenter, Goethe-University Frankfurt am Main, Max-von-Laue-Str. 9, D-60438 Frankfurt am Main, Germany; 2 Lehrstuhl für Theoretische Chemie, Ruhr-University Bochum, D-44780 Bochum, Germany; 3 Max Planck Institute of Biophysics, Max-von-Laue-Str. 3, D-60438 Frankfurt am Main, Germany; 4 Cluster of Excellence–Macromolecular Complexes, Goethe-University Frankfurt am Main, Max-von-Laue-Str. 9, D-60438 Frankfurt am Main, Germany

## Abstract

Salt bridges in lipid bilayers play a decisive role in the dynamic assembly and downstream signaling of the natural killer and T-cell receptors. Here, we describe the identification of an inter-subunit salt bridge in the membrane within yet another key component of the immune system, the peptide-loading complex (PLC). The PLC regulates cell surface presentation of self-antigens and antigenic peptides via molecules of the major histocompatibility complex class I. We demonstrate that a single salt bridge in the membrane between the transporter associated with antigen processing TAP and the MHC I-specific chaperone tapasin is essential for the assembly of the PLC and for efficient MHC I antigen presentation. Molecular modeling and all-atom molecular dynamics simulations suggest an ionic lock-switch mechanism for the binding of TAP to tapasin, in which an unfavorable uncompensated charge in the ER-membrane is prevented through complex formation. Our findings not only deepen the understanding of the interaction network within the PLC, but also provide evidence for a general interaction principle of dynamic multiprotein membrane complexes in immunity.

Antigen presentation to cytotoxic T-lymphocytes by major histocompatibility complex class I (MHC I) molecules is a major process in adaptive immunity. The status of every nucleated cell is monitored by cytotoxic T-cells through the presentation of MHC I loaded with peptides, derived from proteasomal degradation products^1^. The peptide-loading complex (PLC) orchestrates peptide recognition and transport as well as the efficient loading of high-affinity peptides onto MHC I molecules. The PLC consists of the heterodimeric transporter associated with antigen processing (TAP1 and TAP2) as its centerpiece, the oxidoreductase ERp57, the lectin-like chaperone calreticulin, the MHC I heavy chain/β_2_-microglobulin (β_2_m) dimer, and the MHC I-specific chaperone tapasin^1^,^2^,^3^,^4^. TAP, for its part, translocates proteasomal degradation products from the cytosol into the lumen of the endoplasmic reticulum (ER), where these peptides are loaded onto MHC I molecules. Peptide-MHC I complexes then travel via the secretory pathway to the cell surface to present their antigenic cargo to cytotoxic T-cells.

Tapasin, a type I membrane glycoprotein, is a key component within the PLC, which links the two main subcomplexes and functional modules of the PLC, namely peptide donor (TAP1/2) and acceptor (MHC I), by binding simultaneously to TAP and MHC I^5^,^6^. Moreover, tapasin is not only a structural adaptor, but also an essential catalyst for the effective loading and editing of MHC I with high-affinity peptides^7^,^8^. Several regions in the ER-lumenal N- and C-terminal IgG-like domain of tapasin have been identified to contribute to the interaction with the α2 and α3 domains of MHC I^9^,^10^,^11^,^12^. In spite of some understanding of the function of the ER-lumenal domains, we have to date only a limited knowledge of the molecular recruitment mechanism of tapasin into the PLC.

The single transmembrane domain (TMD) of tapasin and the N-terminal transmembrane domains of TAP1 and TAP2, named TMD0^TAP1^ and TMD0^TAP2^, are involved in a stoichiometric interaction^13^,^14^. Despite sharing only 17% sequence identity^14^,^15^, both TMD0s adopt a four-helix topology^16^,^17^. Apparently, both domains fulfill the same function and are targeted to the ER membrane independently of the coreTAP transporter, consisting of the remaining 2 × 6 transmembrane helices (TMs), each followed by a nucleotide-binding domain^18^. Despite the pivotal role of the TMD0^TAP1/2^ in PLC assembly, their atomic structures and interaction sites for tapasin binding are not yet determined. For tapasin, a few residues in its TMD were described to be involved in TAP binding^19^,^20^. Notwithstanding the importance of the peptide-loading complex in adaptive immunity, it remains open how the tapasin-MHC I subcomplex is recruited to the central TAP transporter.

Here, we delineate an ionic lock-switch mechanism for the TAP-tapasin interaction. A conserved salt bridge in the ER membrane determines the specifity of the interaction, the PLC assembly, and the overall process of antigen processing. In addition, molecular modeling and all-atom molecular dynamics (MD) simulations provide information on the structural organization of this essential protein-protein interface in the membrane.

## Results

### An Aspartate Residue in the TMD0 of TAP Is Critical for Tapasin Binding

**Figure 1:**
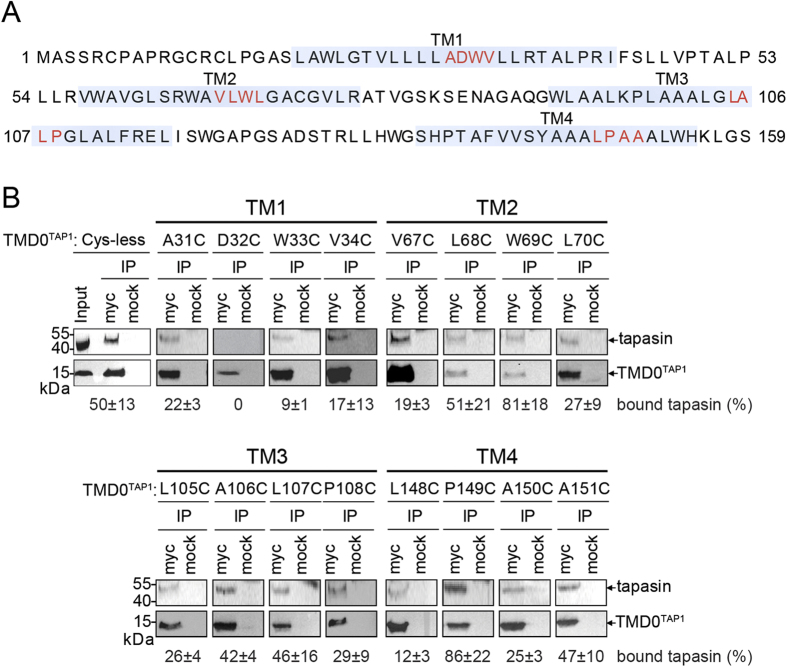
Asp32 in TM1 of TAP1 is essential for tapasin binding. (**A**) Predicted transmembrane helices of human TMD0^TAP1^ are illustrated (light blue). Substitutions of four consecutive residues with cysteine in the center of each TM are marked in red. (**B**) Interaction of the 16 single cysteine mutants with tapasin was probed by co-immunoprecipitation (IP). Representative blots are shown. Densitometric analysis of three independent experiments (mean ± standard error of the mean) of tapasin relative to TMD0^TAP1^ is summarized. Immunoblot signals were quantified by ImageJ (NIH). An isotypic antibody was used as control (mock). The input represents a 1/20 aliquot of the precipitate.

To identify important TAP residues for tapasin binding, we performed a cysteine-scanning mutagenesis approach of TMD0^TAP1^. Analyzing the regions of transmembrane helices in TMD0^TAP1^  (ref. 21) we defined four transmembrane (TM) helices spanning TAP1 residues 20–44 (TM1), 57–76 (TM2), 96–123 (TM3), and 137–160 (TM4) (Fig. 1A). We generated a set of 16 single-cysteine mutants of TMD0^TAP1^ with four consecutive mutations in the middle region of each of the four predicted TMs of Cys-less TAP, which is fully functional in antigen translocation and MHC I loading^22^. Interactions between TMD0^TAP1^ mutants and tapasin were assayed after transient transfection of HeLa cells by co-immunoprecipitation utilizing the C-terminal c-Myc tag of the TMD0^TAP1^ mutants. Immunoblot analysis showed that all single-cysteine mutants were expressed in HeLa cells, albeit at different levels than the wild-type (Fig. 1B). Notably, we found that all single-cysteine TMD0^TAP1^ mutants interact with tapasin, except for the D32C mutant (Fig. 1B). These results suggest an important role for tapasin binding of the aspartate residue at the midpoint of the first transmembrane helix of TAP1.

### A Conserved Salt Bridge Is Essential for TAP-Tapasin Interaction

**Figure 2:**
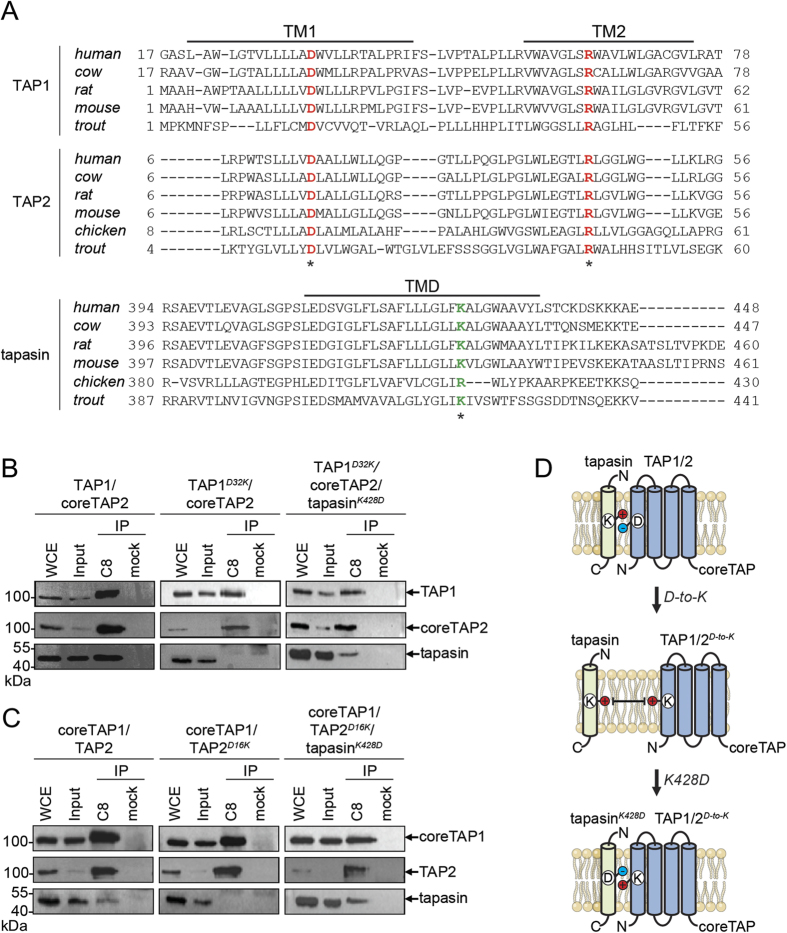
An inter-molecular salt bridge defines the specificity of the TAP-tapasin interaction. (**A**) Sequence alignments for TAP1, TAP2, and tapasin of human, cow, rat, mouse, chicken, and trout are shown. Conserved Asp and Arg residues in TAP and the conserved lysine residue in tapasin are marked in red and green, respectively (asterix). Notably, chicken tapasin has an arginine instead of a lysine and chicken TAP1 has no TMD0. (**B**,**C**) Interaction of full-length human TAP1^*D-to-K*^ and TAP2^*D-to-K*^ with tapasin can be rescued by co-expressing tapasin^*K428D*^ in HeLa cells (charge exchange). TAP complexes fused to mVenus and mCerulean (ref. 24) were selectively purified via tandem affinity purification (IP), and analyzed by SDS-PAGE followed by immunoblotting. Whole cell extract (WCE) and input represent 1/20 aliquot of the precipitate. (**D**) Schematic representation of the TAP-tapasin interaction highlighting the specificity that is determined by an inter-subunit salt bridge in the ER membrane. A *D-to-K* exchange in TAP1/2 interrupts the interaction with tapasin, whereas an additional *K-to-D* exchange in tapasin leads to a rescue.

Despite the low sequence conservation of the entire TMD0, alignments show that D32 in the center of TM1 of TAP1 is conserved (Fig. 2A, Fig. S1). Interestingly, all TAP2 subunits share the same sequence motif, suggesting a common interaction principle for tapasin (Fig. 2A, Fig. S1). To test this hypothesis, we exchanged the conserved aspartate residue for lysine in human, rat, and chicken TAP subunits. Since the human TAP transporter can simultaneously interact with two tapasin molecules^13^,^18^,^23^, we mimicked the avian PLC, in which TAP1 lacks the TMD0, thereby creating asymmetric TAP complexes. Such engineered TAP complexes allow binding of one single tapasin molecule, thereby facilitating the readout of TMD0-tapasin interaction. We have previously shown that full-length human, rat, and chicken TAP1/TAP2 complexes interact with human tapasin^24^. To provide a single binding platform for tapasin, we expressed and purified asymmetric, heterodimeric TAP complexes harboring only one TMD0 either at TAP1 or TAP2. By co-immunoprecipitation, we first confirmed that these asymmetric TAP complexes of human, rat, and chicken origin recruit human tapasin (Fig. 2B,C *left* and Fig. S2*A-C left*). We next substituted the conserved aspartate with lysine (*D-to-K*) in TAP1 and TAP2 and expressed different combinations of these *D-to-K* mutants and coreTAP1 or coreTAP2 to test tapasin binding. Strikingly, all combinations of TAP1^*D-to-K*^ or TAP2^*D-to-K*^with the corresponding coreTAP subunit did not show any interaction with human tapasin, corroborating the essential role of the conserved aspartate residue for the TAP-tapasin interaction (Fig. 2B,C *middle* and Fig. S2*A-C middle*). Finally, we engineered a tapasin mutant in which the conserved K428 is exchanged for an aspartate (tapasin^*K428D*^), and we tested its recruitment to asymmetric, heterodimeric TAP complexes (Fig. 2B,C *right*). Remarkably, this charge conversion recovered the specific interaction for all tested combinations of TAP1^*D-to-K*^ and TAP2^*D-to-K*^ mutants with the corresponding coreTAP subunit. Similar results were obtained across all species investigated (Fig. S2*A-C right*). Taken together, our data demonstrate that a single, conserved inter-subunit salt bridge formed by an aspartate in the first transmembrane helix of TAP1 or TAP2 and a lysine in the single transmembrane domain of tapasin is necessary for the TAP-tapasin interaction (Fig. 2D).

### Antigen Presentation Depends on the Salt Bridge

**Figure 3:**
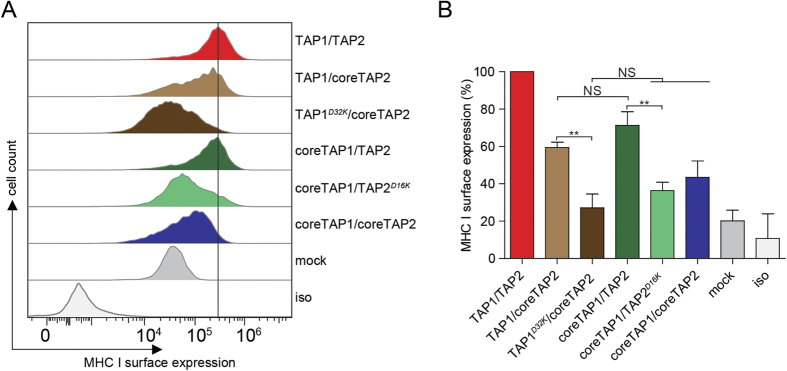
Efficient antigen presentation depends on a membrane-embedded inter-subunit salt bridge in the PLC. (**A**) After nucleofection of TAP-deficient T2 cells with different combinations of TAP subunits as indicated, surface expression of MHC I was monitored by flow cytometry using the PE-coupled anti-human HLA-A/B/C antibody (W6/32). The horizontal line indicates surface expression mediated by TAP1/2. (**B**) Quantification of the MHC I surface expression (**P < 0.05, n = 3). Error bars indicate standard deviations. Statistical analysis was performed using t-test or one-way ANOVA. NS: not significant; mock; transfection with empty plasmid; iso: isotype control.

Effective antigen presentation via MHC I depends on a correct assembly of PLCs possessing at least one tapasin and one MHC I molecule^13^,^23^. The TAP-tapasin interaction is essential for efficient antigen processing, since tapasin acts as an adaptor that keeps MHC I in close proximity to TAP^5^,^13^,^24^,^25^. To check if the *D-to-K* mutations in human TAP1 or TAP2 affect PLC assembly *in vivo*, TAP1/TAP2-deficient T2 cells were nucleofected with combinations of coreTAP1/2 plus TAP1/2 or TAP1/2^*D-to-K*^ to engineer asymmetric TAP complexes with only one tapasin binding site provided by either full-length TAP1 or TAP2. To test the functionality of these TAP1/2 complexes, we monitored the surface expression of peptide-MHC I complexes by flow cytometry. As expected, T2 cells show very low MHC I surface expression because of the defect in antigen translocation, whereas T2 cells nucleofected with TAP1/TAP2 display a strong increase in MHC I surface expression (Fig. 3A,B). Similar results were obtained for asymmetric TAP complexes, corroborating the notion that only one tapasin molecule bound to either TAP1 or TAP2 is sufficient for efficient MHC I antigen loading and presentation^13^,^23^. Notably, PLCs containing either a TAP1^*D-to-K*^ or TAP2^*D-to-K*^mutant showed a defect in MHC I surface expression to a level comparable to coreTAP, indicating impaired PLC assembly and antigen presentation (Fig. 3A,B). These data prove that formation of the conserved transmembrane salt bridge between TAP and tapasin is necessary for efficient peptide loading and surface expression of peptide-MHC I complexes.

### Tapasin Interaction Interface Probed via Cysteine Accessibility

**Figure 4:**
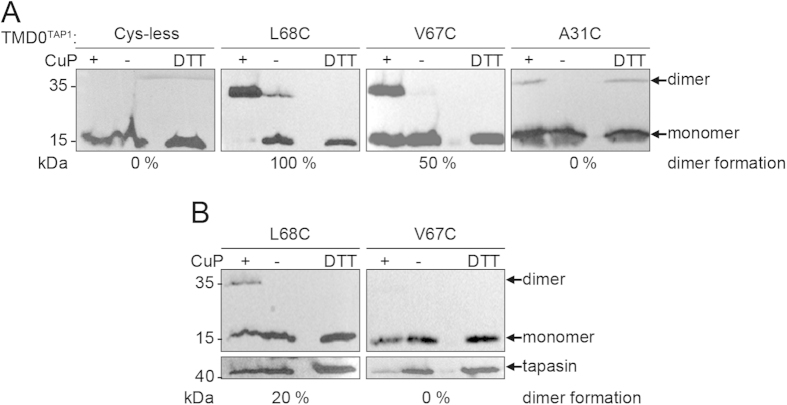
Surface-accessible residues in TMD0^TAP1^. (**A**) Single-cysteine mutants of TMD0^TAP1^ vary in their tendency to form dimers. All 16 cysteine mutations are summarized in Table 1 and Figure S2. (**B**) Decrease in dimer formation in the presence of tapasin. Membranes were prepared from transiently transfected M553 cells, treated with CuP (+), PBS (−), or DTT and subsequently subjected to non-reducing SDS-PAGE and immunoblotting. An empty lane separates the oxidized and reduced sample.

Probing cysteine residues with sulfhydryl-reactive compounds is a common approach to analyze surface accessibility of residues in membrane proteins. However, probing surface-exposed cysteines of TMD0^TAP1^ in complex with tapasin is difficult, since small thiol-specific probes can react with engineered cysteines even within clefts of the 4-TM bundle. Since tapasin forms SDS-insoluble aggregates upon oxidation by copper phenanthroline (CuP), the tapasin-TAP interaction could not be examined by cysteine cross-linking. Based on our unexpected observation that single-Cys mutants of TMD0^TAP1^ showed formation of disulfide cross-linked dimers, we probed the TMD0^TAP1^ architecture and TMD0^TAP1^-tapasin interface by oxidative cysteine cross-linking using CuP. Tapasin-negative M553 cells were co-transfected with the single-Cys TMD0^TAP1^ mutants with or without tapasin. Formation of cross-linked dimers was analyzed by non-reducing SDS-PAGE and immunoblotting. In the absence of tapasin, 12 of the 16 engineered cysteine residues yielded cross-linked dimers to different extends, whereas mutants A31C, D32C, P108C, and P149C did not cross-link (Fig. 4A, Fig. S3, and Table 1). We noticed that proline-to-cysteine mutations led to protein aggregation upon oxidation, indicating that exchange of these residues affects the overall structural integrity of the TMD0^TAP1^. Notably, co-expression of tapasin in M553 cells blocked the cysteine accessibility of seven out of the 16 single-Cys mutants (Fig. 4B, Fig. S3 and Table 1). Four of these blocked residues were located in the predicted helix TM2 (V67C, L68C, W69C, and L70C), two in TM1 (W33C and V34C), and one in TM3 (L107C). Interestingly, all of the mentioned residues are of hydrophobic nature. We therefore conclude that the tapasin interaction interface is centered around TM1 and TM2 of TMD0^TAP1^ with a putative minor interface on TM3.

### Ionic Lock-Switch Mechanism

**Figure 5:**
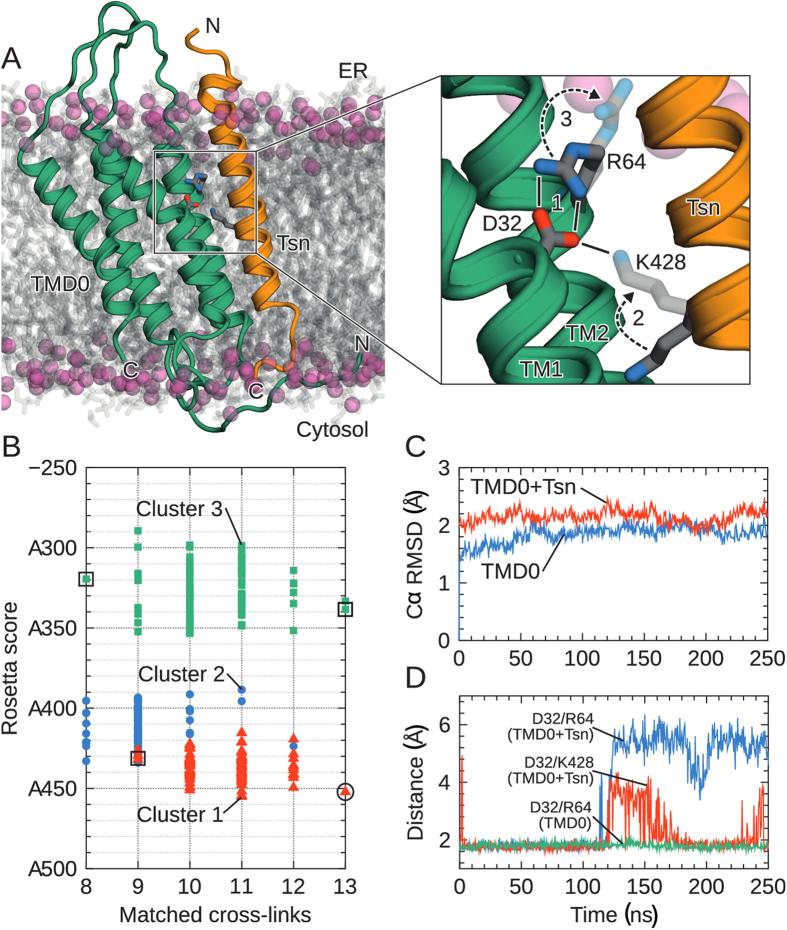
Structure of TMD0^TAP1^ and tapasin-binding mechanism. (**A**) Structure of the TMD0 4-TM bundle associated with the tapasin TM helix (coordinates after 50 ns of MD simulation). Lipid head groups (phosphorus atoms) are shown as spheres; acyl tails are in grey. Water is omitted for clarity. Magnification highlights the ionic lock-switch mechanism: Intra-molecular D32/R64^TAP1^ ionic lock initially stabilizes charged D32 in the membrane (step 1). Upon tapasin binding, the inter-molecular D32/K428^tapasin^ salt bridge is established (step 2). This promotes rupture of the D32/R64 interaction, with R64 moving towards the lipid head groups (step 3). (**B**) Rosetta scores of predicted TMD0 structure clusters and their match to the experimental cross-links, where low scores are favorable. The circle indicates the selected final TMD0^TAP1^ model. Models surrounded by a rectangle were chosen for control simulations (Fig. S4). (**C**) Cα-RMSD of the TM helices with respect to starting structure during MD. (**D**) Time series of the salt bridge (minimal residue-residue distance) illustrating the ionic lock-switch mechanism.

To propose a structural model of TMD0^TAP1^, we assembled four individual TM-helices predicted from multiple sequence alignments using a coarse-grained replica-exchange Monte Carlo approach^26^,^27^. Structural clustering yielded three distinct ensembles (Fig. 5). Based on the coarse-grained structures, we constructed all-atom models with Modeller^28^ and relaxed them using Rosetta^29^. The highest-scoring ensemble (cluster 1) displays a clockwise topology of the four TM-helices (as viewed from the cytosol). A structure of low Rosetta energy within this cluster matched 13 of the 14 experimental cross-links (Table 1 and Fig. S3). This model was embedded into a POPC lipid membrane and subjected to MD in explicit solvent. Two 250-ns simulations were carried out: one of the isolated TMD0^TAP1^ and another of the TMD0^TAP1^ associated with the TMD of tapasin (Fig. 5). The structures were found to be stable on the simulation timescale: the Cα-RMSD of the TM helices plateaus at ~ 2 Å (Fig. 5), and the α-helical content of the entire TMD0 fluctuates around the initial value of 53% (85/160 residues). Much larger structural deviations (helix Cα-RMSD ~3 Å, partial unfolding of α-helices) were observed in three control MD simulations (Fig. S4). The structures selected for these control simulations cover the range from low to high Rosetta energy as well as from few to many matched experimental cross-links (boxes in Fig. 5B). The large conformational changes seen in these control simulations suggest that incorrect structures can be recognized on the 250-ns timescale, though the identification of more subtle issues, such as suboptimal side-chain packing, may require even longer runs.

MD simulations of the TMD0-tapasin complex show concerted changes in amino acid contacts as a result of TMD0-tapasin binding. Figure 5 illustrates the two key salt bridges that are involved in the specific TAP-tapasin interaction forming an ionic-lock relay. In the absence of tapasin, a highly stable intra-molecular salt bridge is formed between D32 of TM1 and R64 of TM2, stabilizing the negative charge buried in the hydrophobic membrane environment. Upon tapasin binding, this interaction is broken and replaced by a new, inter-molecular salt bridge between D32 and tapasin K428 (Supporting Movie S1). We propose that this ionic lock-switch mechanism fulfills two functions: the D32/R64 salt bridge stabilizes individual TMD0 interaction hubs in the absence of tapasin (Fig. S5), whereas the D32 TMD0^TAP1^ / K428 tapasin interaction is crucial for the specific recruitment of tapasin. All key players in this mechanism are highly conserved. Like the previously discussed K428 and D32, R64 is invariant, being present in the TMD0 of TAP1 and TAP2. Furthermore, an additional MD simulation of the charge exchange complex, i.e. D32K in TMD0 and K428D in tapasin, showed that the swapped salt bridge is also stable on the 250-ns time scale (Fig. S6), in line with the experimental observation that the TMD0-tapasin interaction is rescued by this double mutation. Taken together, our simulations suggest a plausible structural model for the charge stabilization mechanism in the ER membrane.

## Discussion

Interhelical and inter-subunit salt bridges play an important role in transmembrane communication and protein networks^30^,^31^,^32^, although positioning polar or charged amino acids into the hydrophobic lipid environment of a membrane is energetically highly unfavorable^33^. This energetic penalty for charged side chains can be partially compensated by the formation of salt bridges^34^. Furthermore, it was suggested that side chains of polar or charged residues located in TM-helices compensate the energetic penalty by snorkeling towards the polar head groups of the lipid bilayer^35^,^36^,^37^. Interestingly, ionizable residues were reported to be indispensable for subunit clustering in the case of the T-cell receptor (TCR)^31^ and the natural killer cell receptor (NKG2D)^30^. In G protein-coupled receptors (GPCRs), charged residues are crucial for receptor activation, ligand specificity, and conformational switching^32^,^38^.

In this study, we delineate an ionic lock-switch mechanism underlying the interaction between the TAP transporter and tapasin. We propose that two highly conserved residues within the TMD0s of TAP1/2 –an aspartate in TM1 and an arginine in TM2– form an intra-molecular salt bridge in the ER membrane and thereby stabilize the TAP subunits in the absence of the binding partner tapasin. Upon tapasin binding, a lysine residue within the TMD of tapasin replaces the arginine side chain, which in turn moves towards the polar head groups of the lipid bilayer (Fig. 5, Supporting Movie S1). Our studies demonstrate that the salt bridge between TMD0 and tapasin is necessary for complex formation and, accordingly, the ionic residues involved are conserved among different species. TM-TM interactions are typically promoted by a GxxxG motif^39^,^40^, polar residues^41^,^42^, or more complex patterns such as Ser/Thr clusters^43^ or QxxS-motifs^44^,^45^. With the exception of the conserved salt bridge, none of these motifs are found in TAP or tapasin. An FxxxFxxxGxxKxxxW motif in the TMD of mouse tapasin was suggested to impact the interaction with TAP^20^. Indeed, our structural modeling and MD simulations position these critical residues into the interaction interface between TAP and tapasin, suggesting an additional stabilizing effect by the FxxxFxxxGxxKxxxW motif. The central lysine in the TMD of tapasin has been identified as a functionally important residue, however its TAP-stabilization effect has only been observed in mice^19^,^46^. Our data demonstrate a central role of this lysine residue for the interaction with TAP.

Components of the PLC are independently inserted into the ER membrane via the Sec61 pathway; hence, stabilization of each component must be ensured before complex assembly. The two charged residues within the TAP-TMD0s are neutralized by our proposed intra-molecular ionic-lock, while the stabilization of the positively charged lysine residue in tapasin is presumably based on its position within the TMD. The charge of the protonated amino group of tapasin-K428 may be further compensated by shielding water molecules and a snorkeling effect^33^,^47^. As observed, mutation of D32 leads to significantly lower expression levels of TMD0^TAP1^ compared to the other single-Cys mutants, indicating a stabilizing effect of the ionic-lock on the TMD0^TAP1^ in the absence of tapasin. We demonstrate that mutation of the aspartate residue in the first TM of the TMD0s completely abolishes the TAP-tapasin interaction and that rescue only occurs via a charge exchange. In contrast, mutation of K428 in tapasin only leads to an impaired but not complete loss of the interaction with TAP^19^. The dramatic effect of the aspartate substitution is likely due to the removal of the negatively charged side chain. This modification disrupts the intra-molecular salt bridge and the TMD0-tapasin ionic-lock, thus leading to destabilization.

The spatial proximity between TAP and MHC I promoted by tapasin is crucial for an effective MHC I surface expression, as shown by experiments using tapasin lacking its TMD^13^,^25^. Such soluble tapasin variants could only partially restore MHC I surface expression. When restoring MHC I surface expression in TAP-deficient T2 cells by TAP mutants lacking the ability to bind tapasin, we observed similar effects as for soluble tapasin, further supporting the importance of the TAP-tapasin-MHC I interplay in adaptive immunity.

Here, we identified by a cysteine scanning approach a number of residues in TMD0^TAP1^ that display distinct sulfhydryl reactivity upon tapasin binding. Loss of the positively charged lysine residue of tapasin can presumably be compensated by the hydrophobic interface between TAP and tapasin. In conclusion, our findings unravel a key interaction principle in the formation of the peptide loading complex necessary for an effective immune response.

## Methods

### DNA Constructs

Plasmids coding for human, rat, and chicken full-length and coreTAP as well as for tapasin and TMD0 were described previously^18^,^24^. TAP1 constructs contain a C-terminal C8 epitope (PDRPEG) followed by a His_10_ tag, whereas TAP2 constructs encode a C-terminal c-Myc tag and streptavidin-binding peptide (SBP). TMD0s contain a C-terminal c-Myc tag. Mutants were generated using the QuickChange procedure (Stratagene) and verified by DNA sequencing.

### Cell Lines

All cell types used in this study were cultured at 37 °C in a humidified atmosphere with 5% CO_2_. HeLa cells were cultured in DMEM (Gibco) supplemented with 10% fetal calf serum (FCS). The tapasin-deficient human melanoma cell line M553 and the TAP-deficient T2 cell line were maintained in RPMI 1640 media (Gibco) supplemented with 10% FCS.

### Transfections

HeLa cells were transfected with polyethyleneimine (PEI) in a DNA-to-PEI ratio of one to four. 24 h prior transfection, 5 × 10^6^ cells were seeded in DMEM/10% FCS in 10-cm dishes. 12.5 μg DNA and 50 μg PEI were separately diluted in 700 μl serum-free DMEM. DNA and PEI solutions were mixed, incubated for 30 min at room temperature, and added to the cells while rocking. Media was replaced with fresh DMEM/10% FCS 12 h post transfection. Cells were harvested with a cell scraper and washed with ice-cold PBS 48 h post transfection and subsequently subjected to co-immunoprecipitation experiments. Transient transfection of M553 cells was performed by using XtremeGene HP (Roche). Here, five 15-cm dishes with 2 × 10^7^ cells were used per transfection. Cells were harvested and crude membranes were prepared 48 h post transfection.

### Nucleofection

TAP-deficient T2 cells were nucleofected by using the Amaxa Cell Line Nucleofector Kit C (Lonza, Cologne, Germany), with minor modifications. Briefly, 12 μg DNA was combined with 2 × 10^6^ T2 cells in 100 μl Amaxa Cell Line Nucleofector Kit C solution at room temperature. The mixture was transferred to the supplied cuvettes and nucleofected with program A30 in the nucleofector device. Nucleofected cells were allowed to recover for 24 h before screening for MHC I surface expression by flow cytometry.

### Membrane Preparation

Crude membranes were prepared from confluent 5 × 15-cm dishes of transiently transfected M553 cells. After harvesting the cells with a cell scraper, the cell pellet was resuspended in ice-cold hypertonic lysis buffer (10 mM Tris/HCl, pH 7.5, 0.5 mM MgCl_2_) containing protease inhibitor mix (Serva, Heidelberg, Germany). The cells were dounced using a glass homogenizer and the post-nuclear supernatant was collected by centrifugation (10 min, 4 °C, 700× *g*). Subsequently, the membranes were sedimented by an ultra-centrifugation step (30 min, 4 °C, 100,000× *g*).

### Cysteine Cross-linking

Crude membranes were resuspended in PBS and directly used for oxidative disulfide cross-linking as previously described^48^. Briefly, membranes were incubated for 10 min with 1 mM Cu^2+^(phenanthroline)_3_ (CuP) at 37 °C. Cross-linking was stopped by adding 10 mM of EDTA and *N*-ethylmaleimide (NEM). Membranes were spun down and resuspended in 20 μl SDS sample buffer before being subjected to non-reducing 10% SDS-PAGE and immunoblotting.

### Co-immunoprecipitation

Single-Cys mutants of TMD0^TAP1^ were co-immunoprecipitated with mouse anti-Myc 4A6 antibody loaded sheep anti-mouse Dynabeads (Life-Technologies) for 2 h at 4 °C. Control precipitations were performed using a non-specific isotype-matched mouse antibody. Before, the coated beads were washed with 3 ml IP-buffer (20 mM Tris/HCl, pH 7.4, 0.1% BSA, 150 mM NaCl, and 5 mM MgCl_2_, 1% protease inhibitor mix). Transfected HeLa cells were harvested and solubilized in 1 ml IP buffer containing 1% digitonin for 1 h at 4 °C on a head-over-tail rotator. The cell lysate was centrifuged (30 min, 4 °C, 100,000 × *g*) and the supernatant was pre-cleared using uncoated Dynabeads. The pre-cleared supernatant was incubated with antibody-coated Dynabeads for 1 h at 4 °C. The beads were washed with IP-buffer containing 0.1% digitonin and bound protein was eluted with 30 μl SDS sample buffer. Samples were heated 10 min at 70 °C before analyzing them by SDS-PAGE (10%) followed by immunoblotting.

For immunoprecipitation of *D-to-K* mutants of human, rat, and chicken TAP1 and TAP2, transiently transfected HeLa cells were solubilized as detailed above. A tandem affinity precipitation protocol was used, as previously described^24^. Briefly, HeLa cell lysate was incubated with streptavidin-agarose beads (Pierce) for 1 h at 4 °C. Afterwards, the beads were washed with IP buffer containing 0.1% digitonin. Bound protein was eluted with 1 ml IP-buffer containing 2.5 mM of biotin. Subsequently, eluted protein was added to Dynabeads coated with the C8 antibody and incubated 1 h at 4 °C. After washing, bound proteins were eluted with 35 μl SDS sample buffer and heated for 10 min at 95 °C prior to analysis by SDS-PAGE (10%) followed by immunoblotting.

### Immunoblotting

For immunoblotting, the protein samples were heated for 10 min as indicated. The primary antibodies anti-Myc (TMD0^TAP1^, TAP2) (clone 4A6, Merck Millipore), anti-C8 (TAP1), anti-tapasin (clone 7F6)^18^, or β-actin (clone AC-74, Sigma-Aldrich) were used. Blots were incubated with Clarity Western ECL reagent (BioRad) and chemiluminescence was recorded with a Lumi-Imager (Roche) and analyzed with LumiAnalyst software. The signals of the immunoblots were quantified by using ImageJ (NIH).

### Flow Cytometry

MHC I surface expression of nucleofected T2 cells was monitored by using the monoclonal antibody HLA-A/B/C (W6/32)-PE (BioLegend). Cells were harvested 24 h after nucleofection and washed once with ice-cold FACS buffer (PBS/2% FCS). All further steps were carried out on ice. After blocking with FACS buffer containing 5% BSA, cells were washed twice and incubated with anti-HLA-A/B/C (W6-32)-PE or PE-coupled isotype control antibody for 30 min in the dark. Subsequently, cells were washed and FACS analysis was performed using an Attune acoustic focusing cytometer (Life technologies). Data were analyzed with FlowJo software (TreeStar). Mean fluorescence intensities (MFI) were calculated, TAP1/2 was set on 100% and other transfectants were normalized to the MFI of TAP1/2.

### Statistics

Statistical analysis was performed by GraphPad Prism6. For group analysis, t-test or one-way ANOVA was used.

### Theoretical Methods

TM-helices were determined from a MAFFT multiple sequence alignment of TAP1 sequences. Helices were built with PyMOL. To assemble 4-TM bundle structures, we used coarse-grained replica-exchange Monte Carlo (MC) simulations^26^,^27^ with an adapted residue-pair specific short-range Cα-potential^49^. Each TM-helix was allowed to translate and rotate, without consideration of connecting loops. To enhance sampling, 20 replicas between 100 and 300 K were simulated for 10^6 ^MC steps, keeping 10^4^ configurations at 100 K for the subsequent stage. Structures were clustered according to DRMS. Full-atom models (including loops) were generated from the cluster centers with MODELLER (v.9.13)^28^. Finally, these models were relaxed and rescored using Rosetta (v.3.5)^29^ with an implicit membrane potential^50^. 100 models were produced for each cluster and analyzed according to Rosetta score and surface exposure^51^ of the experimentally cross-linked residues. MD simulations were performed with GROMACS (v.4.6.5)^52^, using the Amber99SB-ILDN force field^53^, POPC lipids^54^, and TIP3P water. NpT ensembles (300 K, 1 bar) were simulated under periodic boundary conditions with PME long-range electrostatics. To generate starting configurations for the MD, pre-oriented proteins^55^ were inserted into a pre-equilibrated bilayer (256 lipids), yielding total system sizes of ca. 60,000 atoms.

## Additional Information

**How to cite this article**: Blees, A. *et al.* Assembly of the MHC I peptide-loading complex determined by a conserved ionic lock-switch. *Sci. Rep.* **5**, 17341; doi: 10.1038/srep17341 (2015).

## Supplementary Material

Supplementary Movie S1

Supplementary Information

## Figures and Tables

**Table 1:**
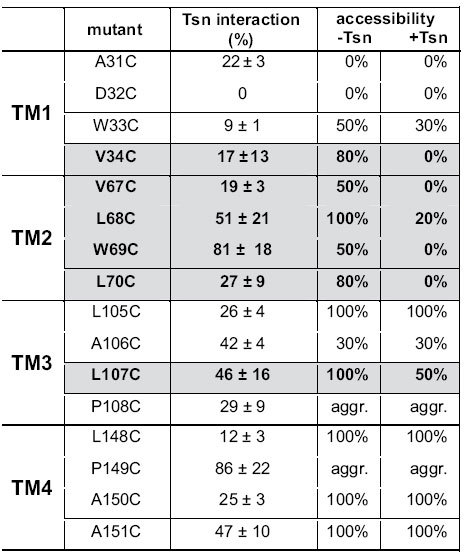
Tapasin interaction and accessibility of single cysteine residues in TMD0^TAP1^ in the presence and absence of tapasin.

## References

[b1] Hulpke S. & Tampé R. The MHC I loading complex: a multitasking machinery in adaptive immunity. Trends Biochem Sci 38, 412–420 (2013). 23849087 10.1016/j.tibs.2013.06.003

[b2] Blum J. S., Wearsch P. A. & Cresswell P. Pathways of antigen processing. Annu Rev Immunol 31, 443–473 (2013). 23298205 10.1146/annurev-immunol-032712-095910 PMC4026165

[b3] Neefjes J., Jongsma M. L., Paul P. & Bakke O. Towards a systems understanding of MHC class I and MHC class II antigen presentation. Nat Rev Immunol 11, 823–836 (2011). 22076556 10.1038/nri3084

[b4] Parcej D. & Tampé R. ABC proteins in antigen translocation and viral inhibition. Nat Chem Biol 6, 572–580 (2010). 20644544 10.1038/nchembio.410

[b5] Ortmann B. *et al.* A critical role for tapasin in the assembly and function of multimeric MHC class I-TAP complexes. Science 277, 1306–1309 (1997). 9271576 10.1126/science.277.5330.1306

[b6] Sadasivan B., Lehner P. J., Ortmann B., Spies T. & Cresswell P. Roles for calreticulin and a novel glycoprotein, tapasin, in the interaction of MHC class I molecules with TAP. Immunity 5, 103–114 (1996). 8769474 10.1016/s1074-7613(00)80487-2

[b7] Howarth M., Williams A., Tolstrup A. B. & Elliott T. Tapasin enhances MHC class I peptide presentation according to peptide half-life. Proc Natl Acad Sci U S A 101, 11737–11742 (2004). 15286279 10.1073/pnas.0306294101 PMC511045

[b8] Fleischmann G., Fisette O., Thomas C., Wienecke R., Tumulka F., Schneeweiss C., Springer S., Schäfer L. V. & Tampé R. Mechanistic Basis for Epitope Proofreading in the Peptide-Loading Complex. J Immunol 195, 4503–4513 (2015). 26416272 10.4049/jimmunol.1501515

[b9] Bangia N., Lehner P. J., Hughes E. A., Surman M. & Cresswell P. The N-terminal region of tapasin is required to stabilize the MHC class I loading complex. Eur J Immunol 29, 1858–1870 (1999). 10382748 10.1002/(SICI)1521-4141(199906)29:06<1858::AID-IMMU1858>3.0.CO;2-C

[b10] Dong G., Wearsch P. A., Peaper D. R., Cresswell P. & Reinisch K. M. Insights into MHC class I peptide loading from the structure of the tapasin-ERp57 thiol oxidoreductase heterodimer. Immunity 30, 21–32 (2009). 19119025 10.1016/j.immuni.2008.10.018 PMC2650231

[b11] Simone L. C., Georgesen C. J., Simone P. D., Wang X. & Solheim J. C. Productive association between MHC class I and tapasin requires the tapasin transmembrane/cytosolic region and the tapasin C-terminal Ig-like domain. Mol Immunol 49 628–639 (2012). 22169163 10.1016/j.molimm.2011.11.002 PMC3249531

[b12] Turnquist H. R. *et al.* A region of tapasin that affects L(d) binding and assembly. J Immunol 167, 4443–4449 (2001). 11591770 10.4049/jimmunol.167.8.4443

[b13] Hulpke S., Baldauf C. & Tampé R. Molecular architecture of the MHC I peptide-loading complex: one tapasin molecule is essential and sufficient for antigen processing. FASEB J 26, 5071–5080 (2012). 22923333 10.1096/fj.12-217489

[b14] Koch J., Guntrum R. & Tampé R. The first N-terminal transmembrane helix of each subunit of the antigenic peptide transporter TAP is essential for independent tapasin binding. FEBS Lett 580, 4091–4096 (2006). 16828748 10.1016/j.febslet.2006.06.053

[b15] Koch J., Guntrum R., Heintke S., Kyritsis C. & Tampé R. Functional dissection of the transmembrane domains of the transporter associated with antigen processing (TAP). J Biol Chem 279, 10142–10147 (2004). 14679198 10.1074/jbc.M312816200

[b16] Schrodt S., Koch J. & Tampé R. Membrane topology of the transporter associated with antigen processing (TAP1) within an assembled functional peptide-loading complex. J Biol Chem 281, 6455–6462 (2006). 16407277 10.1074/jbc.M509784200

[b17] Baldauf C., Schrodt S., Herget M., Koch J. & Tampé R. Single residue within the antigen translocation complex TAP controls the epitope repertoire by stabilizing a receptive conformation. Proc Natl Acad Sci USA 107, 9135–9140 (2010). 20439763 10.1073/pnas.1001308107 PMC2889111

[b18] Hulpke S. *et al.* Direct evidence that the N-terminal extensions of the TAP complex act as autonomous interaction scaffolds for the assembly of the MHC I peptide-loading complex. Cell Mol Life Sci 69, 3317–3327 (2012). 22638925 10.1007/s00018-012-1005-6 PMC3437018

[b19] Petersen J. L. *et al.* A charged amino acid residue in the transmembrane/cytoplasmic region of tapasin influences MHC class I assembly and maturation. J Immunol 174, 962–969 (2005). 15634919 10.4049/jimmunol.174.2.962

[b20] Papadopoulos M. & Momburg F. Multiple residues in the transmembrane helix and connecting peptide of mouse tapasin stabilize the transporter associated with the antigen-processing TAP2 subunit. J Biol Chem 282, 9401–9410 (2007). 17244610 10.1074/jbc.M610429200

[b21] Viklund H. & Elofsson A. OCTOPUS: improving topology prediction by two-track ANN-based preference scores and an extended topological grammar. Bioinformatics 24, 1662–1668 (2008). 18474507 10.1093/bioinformatics/btn221

[b22] Heintke S. *et al.* Functional cysteine-less subunits of the transporter associated with antigen processing (TAP1 and TAP2) by *de novo* gene assembly. FEBS Lett 533, 42–46 (2003). 12505156 10.1016/s0014-5793(02)03746-8

[b23] Panter M. S., Jain A., Leonhardt R. M., Ha T. & Cresswell P. Dynamics of major histocompatibility complex class I association with the human peptide-loading complex. J Biol Chem 287, 31172–31184 (2012). 22829594 10.1074/jbc.M112.387704 PMC3438949

[b24] Hinz A. *et al.* Assembly and function of the major histocompatibility complex (MHC) I peptide-loading complex are conserved across higher vertebrates. J Biol Chem 289, 33109–33117 (2014). 25320083 10.1074/jbc.M114.609263 PMC4246071

[b25] Lehner P. J., Surman M. J. & Cresswell P. Soluble tapasin restores MHC class I expression and function in the tapasin-negative cell line. 220. Immunity 8, 221–231 (1998). 9492003 10.1016/s1074-7613(00)80474-4

[b26] Kim Y. C. & Hummer G. Coarse-grained models for simulations of multiprotein complexes: application to ubiquitin binding. J Mol Biol 375, 1416–1433 (2008). 18083189 10.1016/j.jmb.2007.11.063 PMC2343030

[b27] Kim Y. C., Tang C., Clore G. M. & Hummer G. Replica exchange simulations of transient encounter complexes in protein-protein association. Proc Natl Acad Sci USA 105, 12855–12860 (2008). 18728193 10.1073/pnas.0802460105 PMC2529066

[b28] Webb B. & Sali A. Comparative Protein Structure Modeling Using MODELLER. Curr Protoc Bioinformatics 47, 561–563 (2014). 10.1002/0471250953.bi0506s47 25199792

[b29] Tyka M. D. *et al.* Alternate states of proteins revealed by detailed energy landscape mapping. J Mol Biol 405, 607–618 (2011). 21073878 10.1016/j.jmb.2010.11.008 PMC3046547

[b30] Garrity D., Call M. E., Feng J. & Wucherpfennig K. W. The activating NKG2D receptor assembles in the membrane with two signaling dimers into a hexameric structure. Proc Natl Acad Sci USA 102, 7641–7646 (2005). 15894612 10.1073/pnas.0502439102 PMC1140444

[b31] Call M. E., Pyrdol J., Wiedmann M. & Wucherpfennig K. W. The organizing principle in the formation of the T cell receptor-CD3 complex. Cell 111, 967–979 (2002). 12507424 10.1016/s0092-8674(02)01194-7 PMC3420808

[b32] Janovick J. A. & Conn P. M. Salt bridge integrates GPCR activation with protein trafficking. Proc Natl Acad Sci USA 107, 4454–4458 (2010). 20160100 10.1073/pnas.0914261107 PMC2840094

[b33] Hessa T. *et al.* Recognition of transmembrane helices by the endoplasmic reticulum translocon. Nature 433, 377–381 (2005). 15674282 10.1038/nature03216

[b34] Honig B. H. & Hubbell W. L. Stability of “salt bridges” in membrane proteins. Proc Natl Acad Sci USA 81, 5412–5416 (1984). 6591197 10.1073/pnas.81.17.5412 PMC391714

[b35] Bano-Polo M. *et al.* Polar/Ionizable residues in transmembrane segments: effects on helix-helix packing. PLoS One 7 e44263 (2012). 22984481 10.1371/journal.pone.0044263 PMC3440369

[b36] Schow E. V. *et al.* Arginine in membranes: the connection between molecular dynamics simulations and translocon-mediated insertion experiments. J Membr Biol 239, 35–48 (2011). 21127848 10.1007/s00232-010-9330-x PMC3030942

[b37] Walther T. H. & Ulrich A. S. Transmembrane helix assembly and the role of salt bridges. Curr Opin Struct Biol 27, 63–68 (2014). 24907460 10.1016/j.sbi.2014.05.003

[b38] Manglik A. *et al.* Structural Insights into the Dynamic Process of beta2-Adrenergic Receptor Signaling. Cell 161, 1101–1111 (2015). 25981665 10.1016/j.cell.2015.04.043 PMC4441853

[b39] Langosch D., Brosig B., Kolmar H. & Fritz H. J. Dimerisation of the glycophorin A transmembrane segment in membranes probed with the ToxR transcription activator. J Mol Biol 263, 525–530 (1996). 8918935 10.1006/jmbi.1996.0595

[b40] MacKenzie K. R., Prestegard J. H. & Engelman D. M. A transmembrane helix dimer: structure and implications. Science 276, 131–133 (1997). 9082985 10.1126/science.276.5309.131

[b41] Gratkowski H., Lear J. D. & DeGrado W. F. Polar side chains drive the association of model transmembrane peptides. Proc Natl Acad Sci USA 98, 880–885 (2001). 11158564 10.1073/pnas.98.3.880 PMC14678

[b42] Zhou F. X., Cocco M. J., Russ W. P., Brunger A. T. & Engelman D. M. Interhelical hydrogen bonding drives strong interactions in membrane proteins. Nat Struct Biol 7, 154–160 (2000). 10655619 10.1038/72430

[b43] Dawson J. P., Weinger J. S. & Engelman D. M. Motifs of serine and threonine can drive association of transmembrane helices. J Mol Biol 316, 799–805 (2002). 11866532 10.1006/jmbi.2001.5353

[b44] Langosch D. & Arkin I. T. Interaction and conformational dynamics of membrane-spanning protein helices. Protein Sci 18, 1343–1358 (2009). 19530249 10.1002/pro.154 PMC2775205

[b45] Sal-Man N., Gerber D. & Shai Y. The identification of a minimal dimerization motif QXXS that enables homo- and hetero-association of transmembrane helices *in vivo*. J Biol Chem 280, 27449–27457 (2005). 15911619 10.1074/jbc.M503095200

[b46] Simone L. C., Wang X., Tuli A., McIlhaney M. M. & Solheim J. C. Influence of the tapasin C terminus on the assembly of MHC class I allotypes. Immunogenetics 61, 43–54 (2009). 18958466 10.1007/s00251-008-0335-x PMC2706579

[b47] Hessa T. *et al.* Molecular code for transmembrane-helix recognition by the Sec61 translocon. Nature 450, 1026–1030 (2007). 18075582 10.1038/nature06387

[b48] Oancea G. *et al.* Structural arrangement of the transmission interface in the antigen ABC transport complex TAP. Proc Natl Acad Sci USA 106, 5551–5556 (2009). 19297616 10.1073/pnas.0811260106 PMC2657591

[b49] Miyazawa S. & Jernigan R. L. Residue-residue potentials with a favorable contact pair term and an unfavorable high packing density term, for simulation and threading. J Mol Biol 256, 623–644 (1996). 8604144 10.1006/jmbi.1996.0114

[b50] Yarov-Yarovoy V., Schonbrun J. & Baker D. Multipass membrane protein structure prediction using Rosetta. Proteins 62, 1010–1025 (2006). 16372357 10.1002/prot.20817 PMC1479309

[b51] Levy E. D. A simple definition of structural regions in proteins and its use in analyzing interface evolution. J Mol Biol 403, 660–670 (2010). 20868694 10.1016/j.jmb.2010.09.028

[b52] Pronk S. *et al.* GROMACS 4.5: a high-throughput and highly parallel open source molecular simulation toolkit. Bioinformatics 29, 845–854 (2013). 23407358 10.1093/bioinformatics/btt055 PMC3605599

[b53] Lindorff-Larsen K. *et al.* Improved side-chain torsion potentials for the Amber ff99SB protein force field. Proteins 78, 1950–1958 (2010). 20408171 10.1002/prot.22711 PMC2970904

[b54] Cordomí A., Caltabiano G. & Pardo L. Membrane Protein Simulations Using AMBER Force Field and Berger Lipid Parameters. J Chem Theory Comput 8, 948–958 (2012). 26593357 10.1021/ct200491c

[b55] Nugent T. & Jones D. T. Membrane protein orientation and refinement using a knowledge-based statistical potential. BMC Bioinformatics 14, 276 (2013). 24047460 10.1186/1471-2105-14-276 PMC3852961

