# Supplementary material for: Assembly of the MHC I peptide-loading complex determined by a conserved ionic lock-switch
Source: Sci Rep. 2015 Nov 27;5:17341. doi: 10.1038/srep17341 (PMC4661472; doi:10.1038/srep17341)
Supplement: Supplementary Information [file srep17341-s2.pdf]

Supporting Information

Assembly of the MHC I peptide-loading complex determined by a conserved ionic lock-switch

Running title: PLC assembly by a conserved salt bridge

Andreas Blees[1], Katrin Reichel[2,3], Simon Trowitzsch[1], Olivier Fisette[2], Christoph Bock[1], Rupert Abele[1], Gerhard Hummer[3], Lars V. Schäfer[2], and Robert Tampé[1,4,*]

[1]Institute of Biochemistry, Biocenter and [4]Cluster of Excellence–Macromolecular Complexes, Goethe-University Frankfurt am Main, Max-von-Laue-Str. 9, D-60438 Frankfurt am Main, Germany; [2]Lehrstuhl für Theoretische Chemie, Ruhr-University Bochum, D-44780 Bochum, Germany; [3]Max Planck Institute of Biophysics, Max-von-Laue-Str. 3, D-60438 Frankfurt am Main, Germany

[*]To whom correspondence should be addressed. E-mail: tampe@em.uni-frankfurt.de

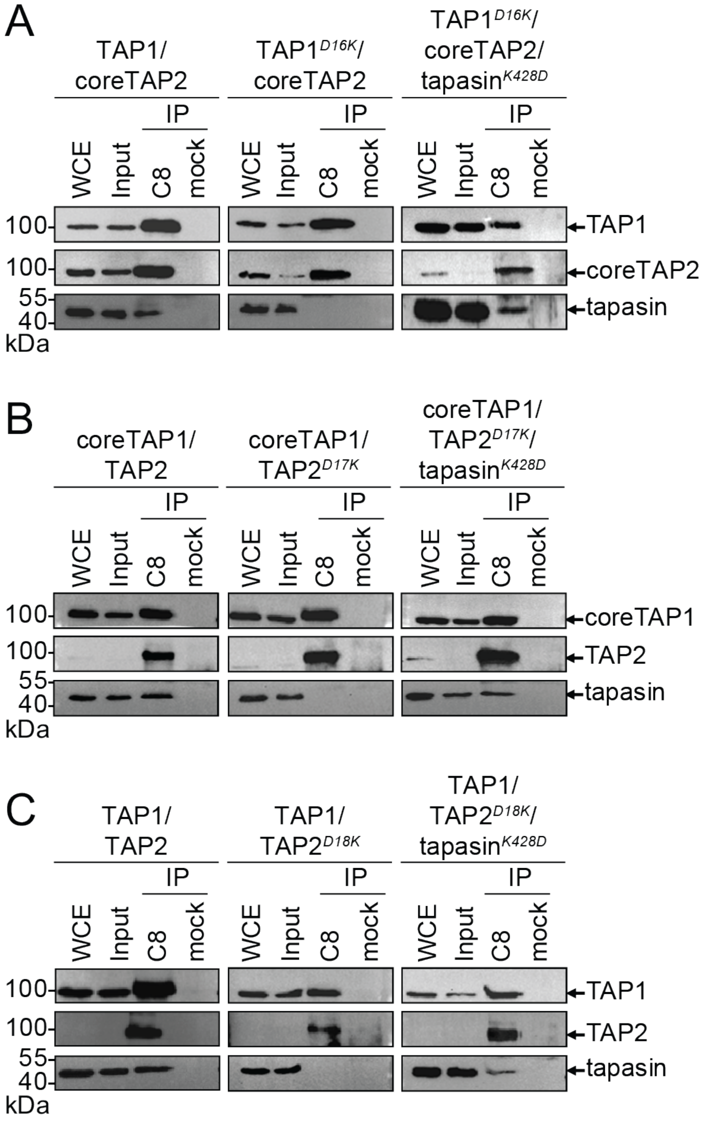

Fig. S1. Charge exchange restores the TAP-tapasin interaction. Interaction *(A-B)* rat and *(C)* chicken TAP1$^{D\text{-}to\text{-}K}$ and TAP2$^{D\text{-}to\text{-}K}$ with tapasin, can be rescued by co-expressing tapasin^{K428D} in HeLa cells. By tandem affinity purification, TAP complexes were selectively purified and subsequently evaluated by SDS-PAGE followed by immunoblotting. Whole cell extract (WCE) and input represent 1/20 aliquot of the precipitate.

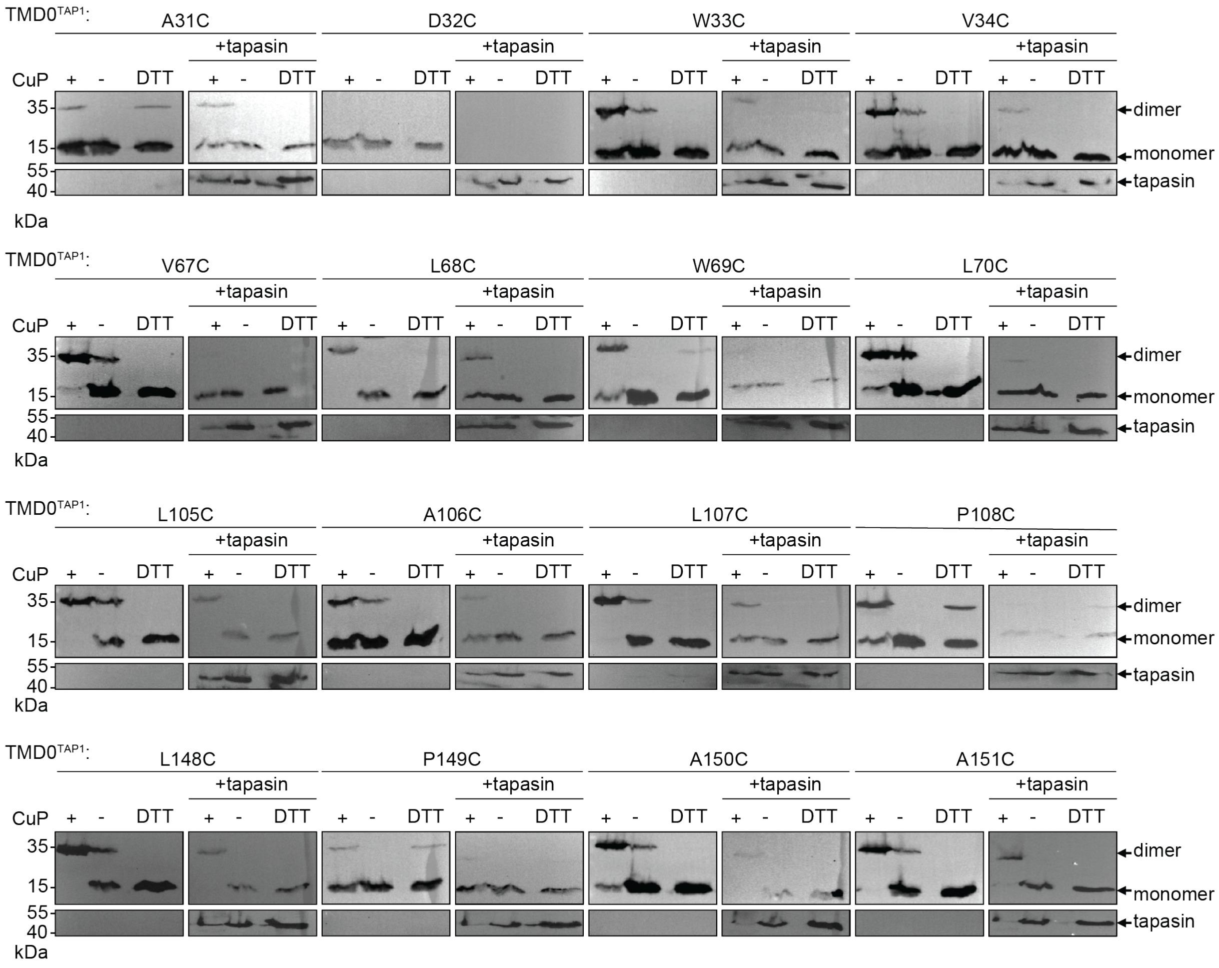

Fig. S2. Surface accessible residues in TMD0^{TAP1} and the effect of tapasin on dimer formation. Membranes were prepared from transiently transfected M553 cells, and treated with CuP (+), PBS (-) or DTT, and subsequently subjected to to non-reducing SDS-PAGE and subsequent immunoblotting. An empty lane separates the oxidative and reduced samples.

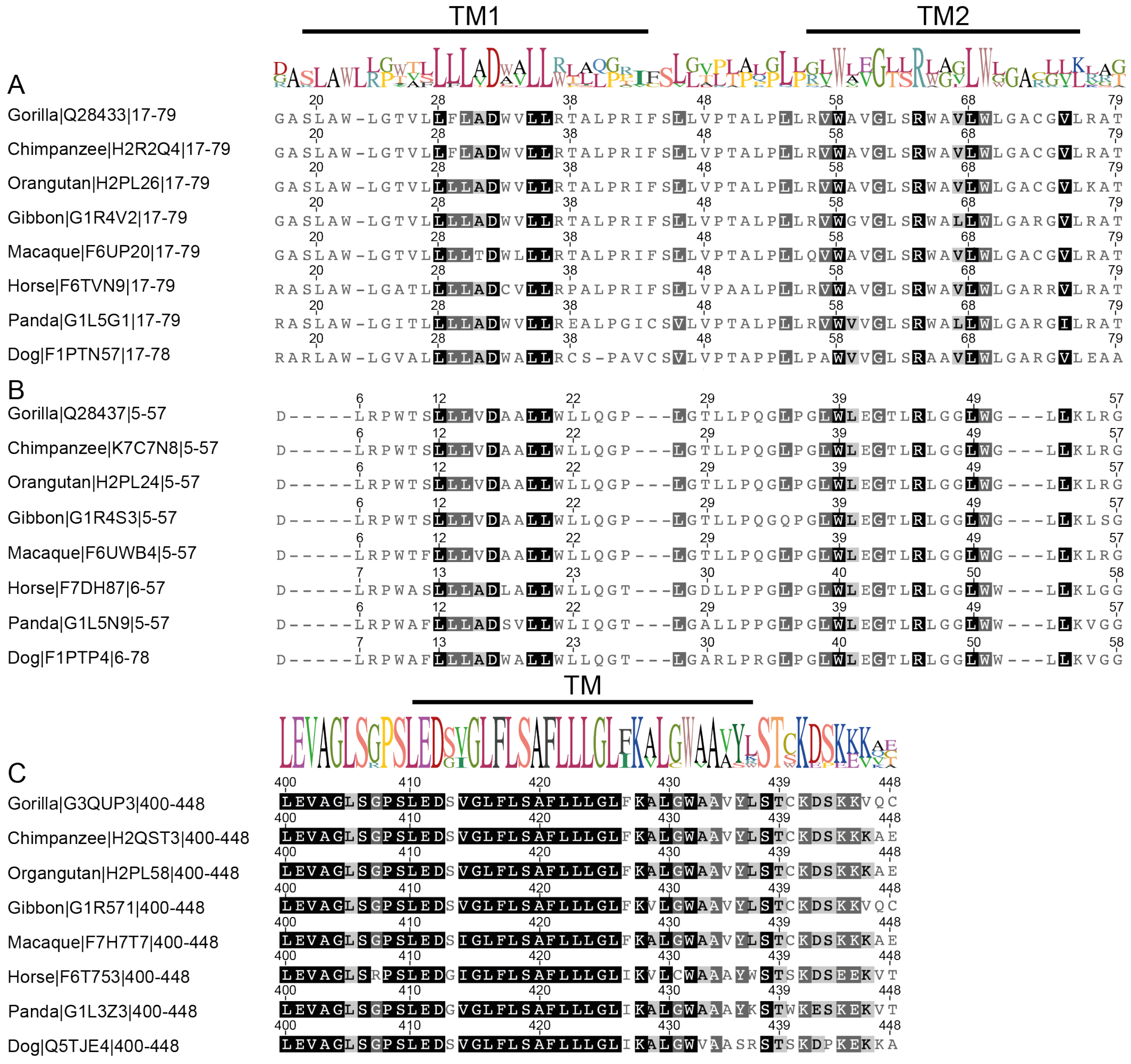

Fig. S3. TAP1, TAP2 and tapasin show conservation of charged residues in their TMs. Multiple sequence alignments (MAFFT) for TAP1 *(A)*, TAP2 *(B)* and tapasin *(C)*. Eight representative species were shown for each protein. Conserved Asp and Arg in TAP1/2 are marked in red, and conserved Lys in tapasin is marked green. TM helices for TAP1/2 and tapasin are indicated.

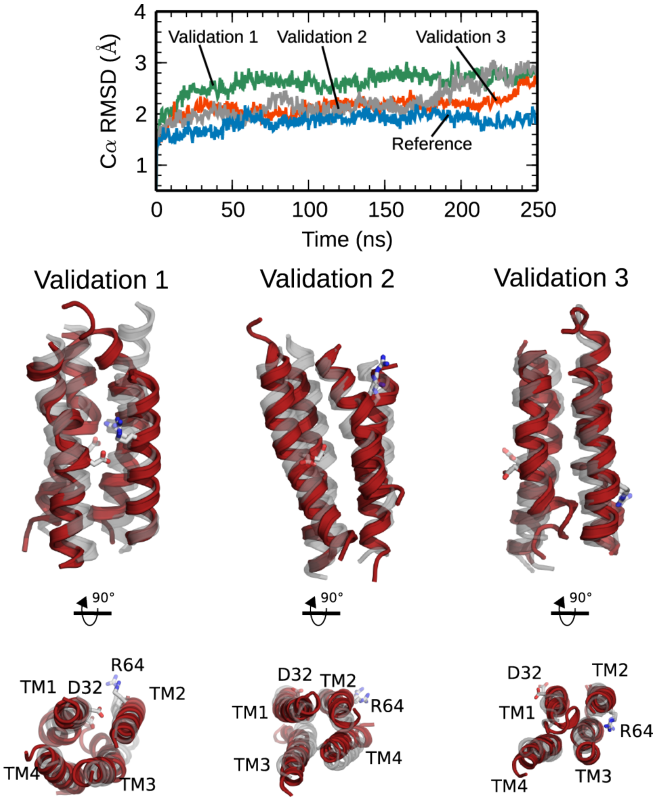

Fig. S4: Control simulations of alternative, lower scoring models. Cα-RMSD time series (TM helices only) with respect to starting structure during MD initiated from (i) model belonging to cluster 1 and matching nine of the thirteen measured cross-links (1, green curve), (ii) cluster 3 model matching eight cross-links (2, grey curve), and (iii) cluster 3 model matching thirteen cross-links (3, red curve). For comparison, the RMSD from Figure 5 (for the top-scoring model) is also shown (blue curve). The structures after 250 ns (red) are superimposed on the starting structure (grey). Side chains of D32 and R64 are shown as sticks.

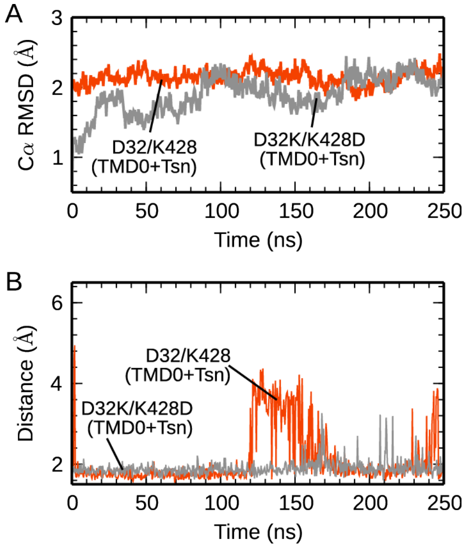

Fig. S5: MD simulation of the charge exchange complex (double mutant TMD0 D32K and tapasin K428D). (A) Cα-RMSD of the TM helices with respect to starting structure. (B) Minimal residue-residue distance shows the stability of the salt bridge on the 250 ns time scale. Grey and red curves show the data for the charge exchange and wild-type systems, respectively.

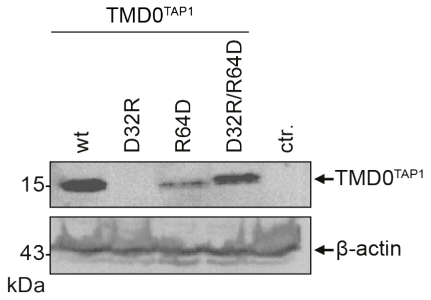

Fig. S6: Charge exchange of the intra-molecular salt bridge leads to stabilization of $TMD0^{TAP1}$. Expression of D32R, R64D and D32R/R64D reveals that the disruption of the intra-molecular salt bridge leads to destabilization of $TMD0^{TAP1}$ (D32R or R64D of human TAP1), whereas charge exchange restores stabilizing effect (D32R/R64D). HeLa cells were transiently transfected

with TMD0^{TAP1} mutants indicated above, and $1x10^6$ cells were loaded per lane. Expression levels were evaluated by SDS-PAGE followed by immunoblotting. Control lane (ctr.) represents untransfected cells

Movie 1: MD simulation of the TAP1-tapasin interaction. The starting structure represents an intermediate state characterized by hydrogen bonds between D32 and both R64 and K428. In the final dimer state, the side-chain of R64 is oriented towards the lipid
